# Image Encryption Scheme with Compressed Sensing Based on New Three-Dimensional Chaotic System

**DOI:** 10.3390/e21090819

**Published:** 2019-08-22

**Authors:** Yaqin Xie, Jiayin Yu, Shiyu Guo, Qun Ding, Erfu Wang

**Affiliations:** Electronic Engineering College, Heilongjiang University, Harbin 150080, China

**Keywords:** chaotic system, compressed sensing, measurement matrix, Arnold scrambling

## Abstract

In this paper, a new three-dimensional chaotic system is proposed for image encryption. The core of the encryption algorithm is the combination of chaotic system and compressed sensing, which can complete image encryption and compression at the same time. The Lyapunov exponent, bifurcation diagram and complexity of the new three-dimensional chaotic system are analyzed. The performance analysis shows that the chaotic system has two positive Lyapunov exponents and high complexity. In the encryption scheme, a new chaotic system is used as the measurement matrix for compressed sensing, and Arnold is used to scrambling the image further. The proposed method has better reconfiguration ability in the compressible range of the algorithm compared with other methods. The experimental results show that the proposed encryption scheme has good encryption effect and image compression capability.

## 1. Introduction

With the rapid development of data network transmission technology, security issues regarding information transmission in communication systems have attracted wide attention from scholars who are devoted to solving them. Today’s society has also turned its focus in this direction. The awareness of cybersecurity protection in all areas of society has generally increased, and research on information security transmission mechanisms has become increasingly crucial [1]. Today, with the continuous growth of transmission bandwidth and data rates, images have become the mainstream form of multimedia information transmission. This is reflected in the fields of remote sensing telemetry and digital watermarking [2,3]; an effective means of ensuring the secure transmission of digital images would be to design a secure and efficient encryption algorithm to encrypt plaintext images, and to ensure that the encryption algorithm is safe, robust, and resistant to attack.

The wide spectrum, noise-like and random characteristics of chaotic systems comprising chaotic-based digital image encryption methods have been widely concerned by many scholars [4]. However, with the increasing demand for digital image transmission, how to reduce the data transmission amount and storage capacity under the premise of ensuring the security, robustness and anti-attack of the encryption method is the key problem in the practical application of the image encryption method. This is also the purpose of this article.

Related research on enhancing image encryption effects focuses on the following aspects: First, the complexity of the chaotic system can be increased through complex chaos or by using chaos with higher dimensions, thereby improving the image encryption effect. Zhou combined two types of one-dimensional chaos to generate a new one-dimensional chaos domain with larger chaotic range and more complex chaotic behavior, and improved the encryption effect of digital images [5]. Chen extended Cat Chaos to 3D and designed a real-time secure symmetric encryption algorithm to solve the problem of fast and high-security image encryption [6]. Mirzae used hyperchaos to design a parallel encryption scheme [7], which improved local encryption robustness. Second, the combination of chaotic systems and other algorithms can increase the complexity of the algorithm and enhance the security of digital images. In this respect, by using chaotic and DNA sequences, Wen used DNA coding and spatiotemporal chaos for encryption [8]. The core idea behind both scholars’ work was to diffuse spatial pixels using chaos. Furthermore, Ye [9] proposed the use of chaotic maps and information entropy for image encryption, thereby avoiding the transformation of pixel positions before traditional diffusion encryption. Liu [10] proposed an S-box non-destructive quantum image encryption scheme, combining linear transformations and nonlinear transformations to improve the complexity of the encryption scheme. Chai [11] shuffled the pixels in digital images by assigning new random access locations to them, which enhanced the sensitivity of plaintext images. Third, minimizing finite precision improves the randomness of chaotic systems and enhances the encryption effect. Li [12] found that some chaotic characteristics degenerate due to the limited precision used in computers. In [13], the author considers finite precision and uses a binary method to deal with the short period of chaos to prevent chaotic degradation; Nardo uses limited precision error to encrypt the image [14]. This method makes the chaotic system have sufficient randomness and improves the encryption effect of the chaotic system.

In recent years, compressed sensing has undergone extremely rapid development. In 2006, Candes [15,16] proposed the theory of compressed sensing. Compared with the Shannon sampling theorem, compressed sensing greatly reduces the sampling rate and computational complexity. As a non-linear technology, compressed sensing can be incorporated into an image encryption scheme, which minimizes the resource occupancy rate of image transmission. In addition, the chaotic system itself has features such as pseudo-randomness and sensitivity, and is often used in the image encryption process [17,18]. Therefore, the combination of compressed sensing and a chaotic system is expected to produce an excellent implementation of compressed image encryption.

Therefore, in order to better enhance the reconstruction effect of compressed images and improve the security of encrypted images, this paper proposes a compressed sensing image encryption scheme based on new three-dimensional chaos. Firstly, the security of a new three-dimensional hyper-chaotic system enhancement algorithm is designed, and the chaotic Lyapunov exponent, bifurcation diagram and complexity are analyzed. Second, in the encryption scheme, a new chaotic system is used as the measurement matrix for compressed sensing, and Arnold is used to scramble the image further. Third, the image compression reconstruction method proposed in this paper is better than other methods. Finally, the security of the encryption algorithm is analyzed, and the proposed encryption algorithm is verified from the aspects of key space, statistical analysis, information entropy and differential analysis, which can resist various attacks.

## 2. New Three-Dimensional Chaotic System and Analysis

### 2.1. New Three-Dimensional Chaotic System

Because the low-dimensional chaotic map structure is simple, its trajectory parameters and initial values are easy to predict, and the commonly used chaotic systems have been widely known by the public [19,20]. Therefore, using existing low-dimensional chaotic signals will threaten the security of image encryption [21]. In contrast, the high-dimensional chaotic map has more variables and parameters, which can make the encryption scheme more secure and the encryption image is more difficult to decipher. For this reason, a new three-dimensional chaotic mapping method was designed. The dynamic equation is as follows:

(1) $$\{\begin{array}{l} x\left(i\right)\;=ax(i-1)+by(i-1)+cz(i-1)+dx(i-1)y(i-1)+ex(i-1)z(i-1)+fy(i-1)z(i-1)\\ y\left(i\right)\;=\;x\left(i-1\right)\\ z\left(i\right)\;=\;y\left(i-1\right) \end{array}$$

**Figure 1:**
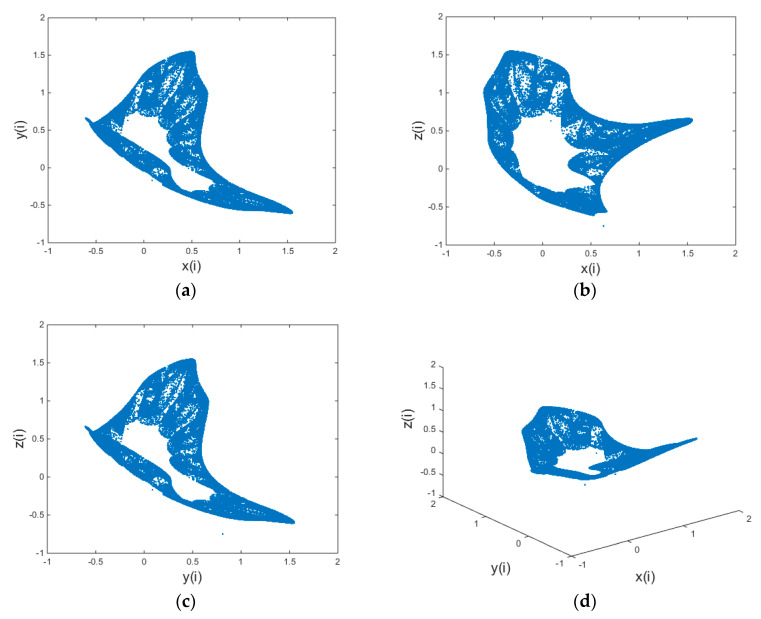
Chaotic attractor; (**a**) x-y plane; (**b**) x-z plane; (**c**) y-z plane; (**d**) perspective view.

The parameter values are: a=−0.54,b=−0.25,c=0.79,d=−1.79,e=−1.69,f=−1.78. When the initial values are $\{\begin{array}{l} x\left(0\right)=0.63\\ y\left(0\right)=0.81\\ z\left(0\right)=-0.75 \end{array}$, the chaotic state can be entered through iteration. The attracctors of the 3D chaotic map are shown in Figure 1.

The Lyapunov exponent is an important indicator that determines whether a system has entered chaos. It qualitatively reflects the sensitivity of chaotic systems to small changes in the initial value, as well as reflecting the local divergence and contraction of trajectories [22]. That is, it reflects the unpredictability and randomness of chaotic trajectories. The definition of the Lyapunov exponent for discrete-time chaotic systems is [23]:

(2) $$\lambda =\underset{n\to \infty }{\lim}\frac{1}{n}\sum_{n=0}^{n-1}\ln\left|\frac{df\left(x_{n},\mu \right)}{dx}\right|$$

**Figure 2:**
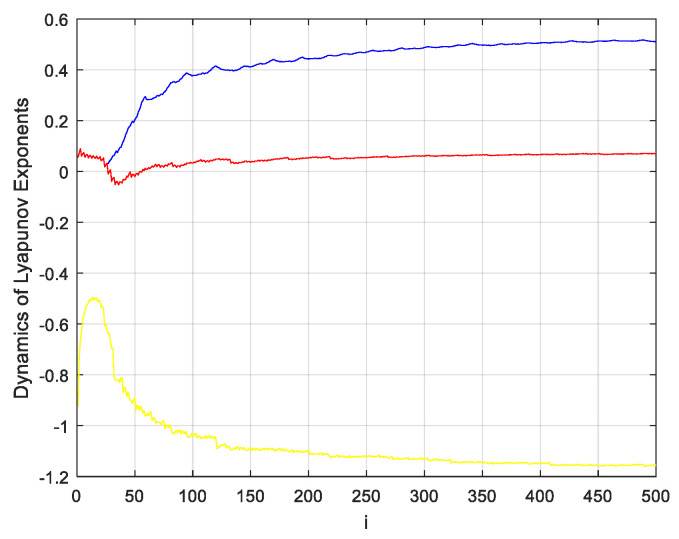
Dynamics diagram of Lyapunov exponents of the new three-dimensional chaotic system.

Based on the Lyapunov exponent theory of chaotic systems [24], the presence of one positive value among the Lyapunov exponents can be taken as an indication that there is chaotic motion; and the greater the number of greater-than-zero Lyapunov exponents present, the greater the complexity of the chaotic motion. If there are two or more positive exponents in a high-dimension phase space, the system may be considered as hyperchaotic. The Lyapunov exponents of the new three-dimensional chaotic system designed in this paper are shown in Figure 2.

**Figure 3:**
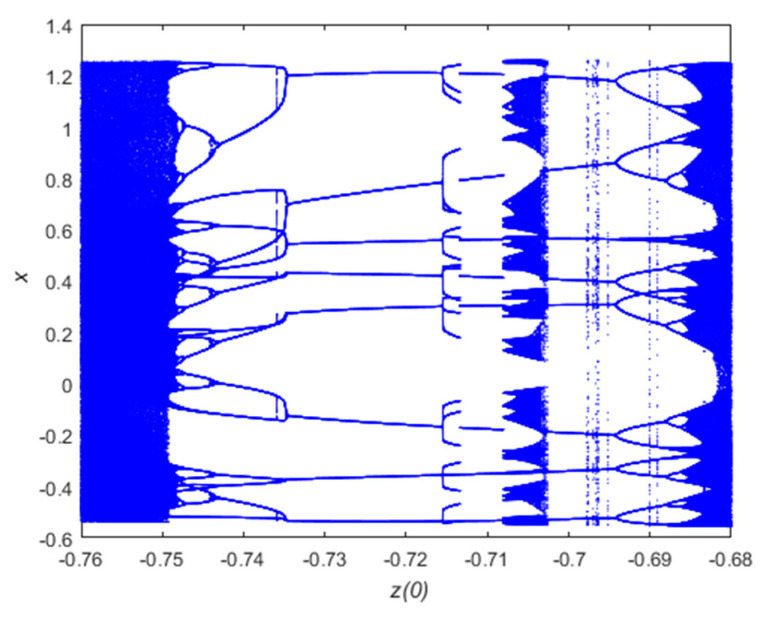
Bifurcation diagram of the new 3D discrete chaotic map.

The bifurcation diagram of a chaotic system is an unstable change behavior caused by a change in parameters [25,26]. If a power system is structurally unstable, small changes can cause sudden changes in the topology of the system. The bifurcation diagram of the chaotic system of this paper is shown below in Figure 3.

The range of values of the initial value z(0) of the chaotic system is changed, and the bifurcation diagram of the chaotic system is shown in Figure 3. With the change of parameters, the bifurcation phenomenon of chaos is obvious to see. On the whole, the iterative sequence generated with larger values is increasingly complicated. It can be seen from the Formula (1) of the three-dimensional dynamic system designed in this paper that the variable z can reflect the iterations of y and x, and will not be described here. 

### 2.2. Complexity Analysis

Approximate entropy represents the complexity of a time series and is a nonlinear dynamic parameter that measures the complexity and unpredictability of sequence fluctuations [27,28]. The main idea is to quantify the time series with a non-negative value. The greater the complexity of the sequence, the larger the corresponding approximation entropy [29]. The specific algorithm for approximate entropy is as follows:

1. Suppose the original data is x(1),x(2),…,x(N), and they are composed of m D vectors in order.
  (3) X(i)=[x(i),x(i+1),…,x(i+m−1)],
  In which i=1,2,3…N−m+1.
2. The distance between x(i) and x(j) is
  (4) $$d(i,j)=\underset{k=1-m-1}{\max}\left[\left|x(i+k)-x(j+k)\right|\right]$$
3. Setting a threshold value r(r>0), for each i, we can obtain the statistics of d(i,j).
  (5) $$C_{i}^{m}(r)=\frac{1}{N-m+1}Sum\left\{d(i,j)<r\right\}$$
4. The mean of logarithm of $C_{i}^{m}(r)$ is written as $\phi^{m}(r)$ and can be calculated by
  (6) $$\phi^{m}(r)=\frac{1}{N-m+1}\sum_{i=1}^{N-m+1}\ln C_{i}^{m}(r)$$
5. Changing dimension and repeating step 1 to step 4, we can obtain the approximate entropy
  (7) $$ApEn(m,r)=\underset{N\to \infty }{\lim}\left[\phi^{m}(r)-\phi^{m+1}(r)\right]$$

However, in practical terms, the length of the data sequence is bounded. Therefore, the approximate entropy algorithm is changed into

(8) $$ApEn(m,r,N)=\phi^{m}(r)-\phi^{m+1}(r)$$

Pincus found that there exists a minimal dependency between ApEn and N when m=2 and r∈[0.1SD(x),0.2SD(x)] [30]. SD(x) is the standard deviation of x. In general, a more complex time series corresponds to a larger entropy value. In general, a more complex time series corresponds to a larger entropy value. It can be seen from Table 1 that under the same parameters, the chaotic sequence of this paper has better complexity and satisfy the requirement of image encryption.

## 3. Compressed Sensing and Scrambling

### 3.1. Compressed Sensing

According to compressive sensing theory, if the signal is sparse or it is sparse in a certain transform domain, then the measurement matrix may be used to project signals onto a low-dimensional space, thereby reducing the required storage capacity [31]. The original information is then reconstructed with high probability using a small number of sampled values projected onto the low dimensional space. In nature, most signals themselves are not sparse, so they must be transformed into other transform domains in order to make them sparse [32]. Assume a finite length signal $X\in R^{N\times 1}$,and get its sparse or near-sparse representation under an appropriate sparse basis $\Psi \in R^{N\times N}$:

*α* = Ψ’*x* (9)

The measurement process of compressive sensing can be mathematically expressed as [33]:

(10) y=Φx=ΦΨα= Θα

where Θ=Φ×Ψ is the sensing matrix; Φ of size M×N(M≪N) is the measurement matrix, so the original signal X cannot be determined directly by observing the value of vector Y. Refactoring x from y requires solving the optimization problem

(11) $$\min{\left\|\alpha \right\|}_{0}s.t.y=\Theta \alpha$$

In (11), α needs to be solved. Typical compressed sensing reconstruction algorithms [34] include the back-propagation (BP) algorithm, the orthogonal matching pursuit (OMP) algorithm, the matching pursuit (MP) algorithm, the stagewise OMP (StOMP) algorithm, and the compressive sampling matching pursuit (CoSaMP) algorithm, among others.

The encryption and decryption algorithm employed in this paper uses measurement matrix and reconstruction algorithms from compressive sensing theory as its core, so this section focuses on the complexity of the CoSaMP reconstruction algorithm. CoSaMP [35,36] combines the main ideas of general combination algorithms to ensure convergence speed and performance. The theorem is as follows. 

Theorem [37]: Suppose that $\Phi \in R^{m\times n}$ is a measurement matrix satisfying the restricted isometry property (RIP) conditions of 2s order, i.e., $\delta_{2s}\leq c$. In this case, y=Φx+w is the measurement of signal *x*, and $x\in R^{n}$ and $w\in R^{n}$ indicate the error terms generated by noise. For a recovery accuracy parameter η, the CoSaMP algorithm can generate a *s*-sparse vector $\hat{x}$ that satisfies:

(12) ‖x−x^‖≥C·max{η,1s‖x−xs2‖1+‖w‖2}

In this formula, xs2 is the approximation of the s2 order sparseness of *x*. The time complexity of the entire algorithm is $O\left(\rho \cdot \mathrm{lg}\left(\frac{{\left\|x\right\|}_{2}}{\eta }\right)\right)$, where ρ is the cost of the multiplication of Φ and $\Phi^{T}$.

### 3.2. Arnold Scrambling

Arnold scrambling is proposed for the research of ergodic theory, is also a kind of image scrambling method based on space position. Arnold scrambling encryption methods are after transform of the image pixel position will rearrange, makes the image look cluttered [38]. A digital image with size M×N can be regarded as a two-dimensional matrix. The pixel locations of the image will rearrange after Arnold transformation, thereby achieving image encryption [39]. The Arnold transform of a two-dimensional digital image of size M×N is defined as:

(13) $$\left[\begin{array}{l} x_{n+1}\\ y_{n+1} \end{array}\right]=\left[\begin{array}{cc} 1 & j\\ i & ij+1 \end{array}\right]\left[\begin{array}{l} x_{n}\\ y_{n} \end{array}\right]\mathrm{mod}(N)$$

where i and j are parameters, n is the number of iterations, and N is the height or width of the image. The inverse-scrambling formula is as follows:

(14) $$\left[\begin{array}{l} x_{n+1}\\ y_{n+1} \end{array}\right]=\left[\begin{array}{cc} ij+1 & -j\\ -i & 1 \end{array}\right]\left[\begin{array}{l} x_{n}\\ y_{n} \end{array}\right]\mathrm{mod}(N)$$

The main reason for encrypting images with Arnold scrambling is that it has periodic characteristics. As the number of iterations increases, the image becomes more chaotic and achieves basic secrecy [40]. At the same time, using Arnold scrambling places certain requirements on the image, and the image to be processed needs to be square.

## 4. Image Encryption and Decryption Schemes

**Figure 4:**
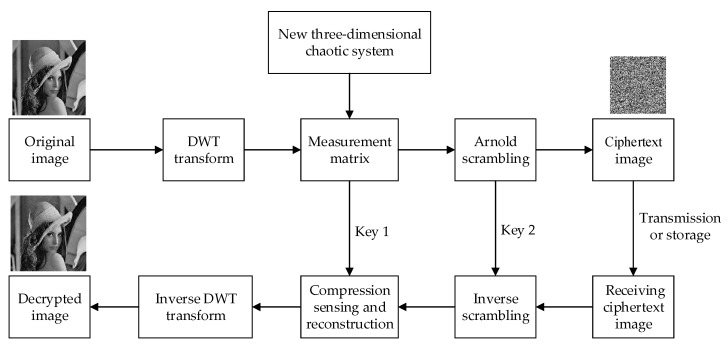
Image encryption and decryption processes.

In this paper, the discrete wavelet transform (DWT) matrix is selected as the sparse matrix. In the decryption process, the CoSaMP algorithm is used for sparse reconstruction. The image encryption and decryption processes are shown in Figure 4.

(1) The initial conditions of the newly designed three-dimensional chaotic system are determined as $\{\begin{array}{l} x\left(0\right)\;=0.63\\ y\left(0\right)\;=0.81\\ z\left(0\right)\;=\;-0.75 \end{array}$, and the parameters are defined as a=−0.54,b=−0.25,c=0.79,d=−1.79,e=−1.69,f=−1.78 in order to iteratively generate the chaotic sequence.
(2) The header data of the chaotic sequence generated by Equation (1) is discarded before the system enters the steady state. The steady state data is retained as the y sequence. It is then reorganized into a measurement matrix Φ of size M×N. The generated compressed sensing measurement matrix is then quantized.
(3) The initial conditions and parameters of the new chaotic image are taken as key 1. That is, the parameters and initial values of the new three-dimensional discrete chaotic system are taken as key 1={a,b,c,d,e,f,x(0),y(0),z(0)}.
(4) DWT is used to make the original image sparse in the wavelet domain, with a sparsity of K=50. Then, two observations are performed on the original image according to the formula $I_{2}=\Phi \cdot {\left(\Phi \Psi I_{1}\right)}^{T}$ to obtain the I_2_ of M×M, where I_1_ is a plaintext image and Ψ is the DWT transformation matrix.
(5) Uniform quantization is performed on I_2_, so that the quantized value is an integer between 0 and 255.
(6) To improve the effect of encryption, the image continues to undergo Arnold scrambling, as per Equation (12). At the same time, the ciphertext image is obtained, and the scrambling parameter and iteration number constitute key 2={i,j,n}.
(7) Decryption is the inverse process of encryption. Key 2 and key 1 are used sequentially to perform inverse Arnold scrambling and inverse DWT transform on the ciphertext image, and finally, compressed sensing and reconstruction using the CoSaMP algorithm is applied to obtain the original image.

## 5. Simulation Experiments and Performance Analysis

### 5.1. Simulation Conditions

**Figure 5:**
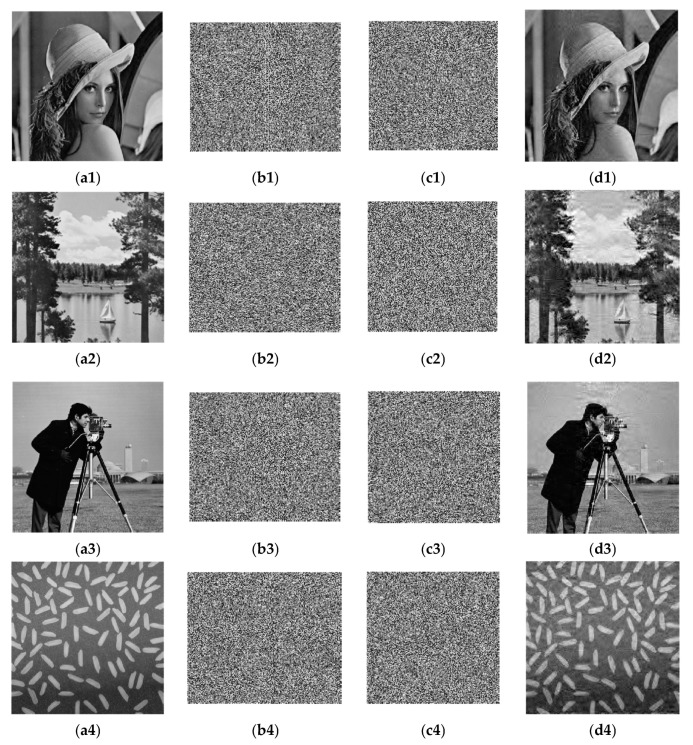
Simulation experiment results. (**a1–a4**) Plaintext images; (**b1–b4**) Images with chaotic encryption; (**c1–c4**) Images with scrambling encryption (ciphertext images); (**d1–d4**) Decrypted images.

In this paper, 256 × 256 grayscale images of Lena, Lake, Cameraman, and Rice are used for testing purposes. Matlab R2010a is used to implement the encryption algorithm. First, the sparsity in the simulation is set as K = 50, and the compression ratio is 0.74, i.e., a 256 × 256 image would be compressed to a 220 × 256 image. After secondary sampling and Arnold scrambling, the ciphertext images are compressed to 220 × 220. The original images, images with chaotic encryption, images with scrambling encryption, and decrypted images are shown in Figure 5.

It can be seen from the simulation results that after a plaintext image passes through the compressed sensing measurement matrix generated by chaos and is scrambled and encrypted, the image size changes, and the ciphertext image completely loses the characteristics of the plaintext image. In terms of visual resolution, the resolution of a decrypted image is lower than the original plaintext image. To accurately evaluate the performance of the compressed image and the security of the encrypted image, detailed analysis is conducted, including algorithm complexity, compression ratio, key space, pictorial diagram analysis, adjacent pixel correlation, information entropy, and resistance to differential attacks.

### 5.2. Compression Ratio

As the image size and decryption algorithm do not change throughout the simulation, the relative complexity of the algorithm also does not change. With the same algorithm complexity, while ensuring the encryption effect of images, the relationship between the image compression rate and the quality of reconstructed images will now be analyzed. In this paper, the image compression ratio is defined as

(15) $$v=\frac{m_{2}\times n_{2}}{m_{1}\times n_{1}}$$

where $m_{1}\times n_{1}$ is the size of a plaintext image, and $m_{2}\times n_{2}$ is the size of a ciphertext image. 

The structural similarity (SSIM) is used to evaluate the accuracy of a reconstructed image [41]. The value of SSIM is between 0 and 1. When two signals are identical, the structural similarity is 1. The SSIM is defined as

(16) $$\mathrm{SSIM}(s,y)=\frac{\left(2\mu_{x}\mu_{y}+c_{1})(2\sigma_{xy}+c_{2}\right)}{\left(\mu_{x}^{2}+\mu_{y}^{2}+c_{1})(\sigma_{x}^{2}+\sigma_{y}^{2}+c_{2}\right)}$$

PSNR is an important indicator for evaluating the quality of decoded images after image processing. It is defined as [42]:

(17) $$PSNR=10\mathrm{lg}\frac{255\times 255}{\left(1/M\times N\right)\sum_{i=1}^{M}\sum_{j=1}^{N}{\left(X\left(i,j\right)-Y\left(i,j\right)\right)}^{2}}$$

where *M × N* is the size of the image, and *X(i, j)* and *Y(i, j)* are the pixel values of the plain image and decrypted image respectively. The larger the PSNR is, the smaller the distortion.

**Figure 6:**
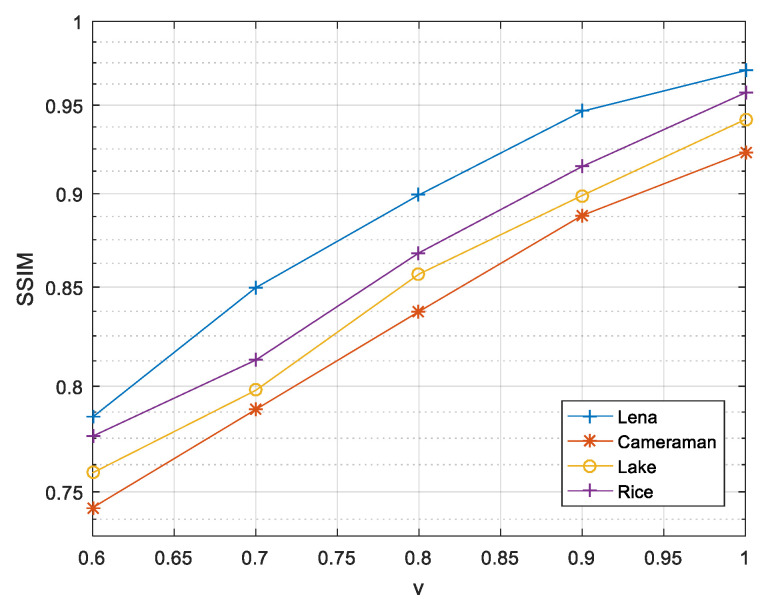
Compression ratios and image reconstruction effects.

The following will analyze the limits of the image compression rate while ensuring the encryption effect and transmission security. In the CoSaMP algorithm, the sparsity is limited to $K<\frac{M}{3}$, so when selecting a sparsity of K = 50, the compression ratio cannot be smaller than 0.6. Therefore, in the compressible range, the image reconstruction effects corresponding to different compression ratios are shown in Figure 6.

It can be seen from Figure 6 that as the compression ratio decreases, the structural similarity coefficient between the reconstructed image and the original image is reduced, but in the compressible range, the reconstruction can be achieved relatively well. Under the same compression ratio and sparsity, the reconstruction effects of different images vary; the reconstruction effect of Lena is significantly better than the other three images.

To compare our compression encryption algorithm with other methods, Table 2 shows that the PSNR value of Lena is reconstructed by different methods in the case of compression ratio v=0.75. 

It can be seen from Table 2 that in the different hyperchaotic systems when the image and compression ratio are the same, the PSNR value of the image recovered by the compression encryption method proposed in this paper is relatively large, so the effect of reconstructing the image is better than other methods.

### 5.3. NIST Test

Pseudo-randomness is an important indicator of the security of encryption algorithms. To further verify the cryptographic characteristics of new chaotic pseudo-random sequences, it is necessary to quantitatively evaluate the pseudo-randomness of cryptographic algorithms [46]. Among the many methods for testing pseudo-randomness, this paper selects sts-2.1.2 [47] in the NIST version. The pseudo-randomness of a sequence is determined by the *p*-value generated by the test results. According to the selected significance level *α*, if *p*-value ≥ *α*, it can be considered to have passed the test. With α=0.01 selected, a 106-bit sequence was taken in the experiment and 100 sequences of this were tested. The test results are shown in Table 3.

The results show that in this sequence of NIST tests, all *p*-values exceed 0.0001, which indicates that the test sequence is uniform. Therefore, the generated sequence is random and this system is suitable for encryption algorithms [48].

### 5.4. Key Space Analysis

Key space analysis is primarily conducted to evaluate the capability of an algorithm to resist exhaustive attacks. For an algorithm with good resistance to exhaustive attacks, the key space must exceed 2^100^ [49,50]. In this paper, there are a total of two encryption algorithm keys; one is composed of the initial values and the parameters of the chaos: key 1={a,b,c,d,e,f,x(0),y(0),z(0)}, with a total of nine parameters; and the other is the scrambling parameter and the number of iterations in Arnold scrambling: key 2={i,j,n}, with a total of three parameters. Since there are 12 parameters in the key space in this paper, it is difficult to accurately locate each parameter. According to the international standard ieee 754, in order to simplify the comparison, the index part is expressed in the form of a positive value. So we calculate the exponent bit of 12 sign bits to about 52 bits, and our key space must be greater than $key_{total}=2^{12\times 52}=2^{624}$.

It can be seen from Table 4 that the proposed algorithm has a key space that is greater than those published in the literature [10,51,52,53]. Therefore, the key space of this algorithm is large enough to resist exhaustive attack.

### 5.5. Analysis of Resistance to Statistical Attacks

#### 5.5.1. Histogram Analysis

**Figure 7:**
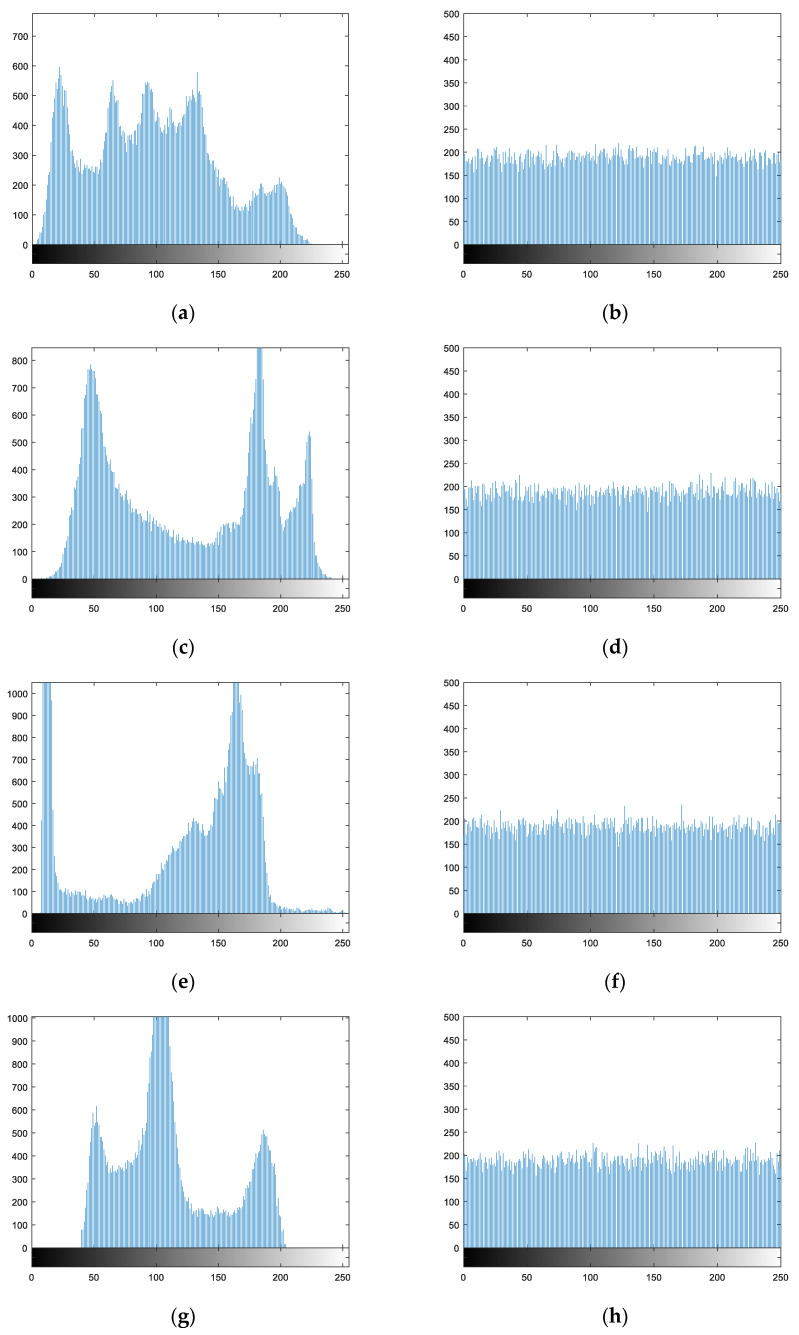
Encryption and decryption histogram analysis: (**a**) plaintext histogram of Lena; (**b**) ciphertext histogram of Lena; (**c**) plaintext histogram of Lake; (**d**) ciphertext histogram of Lake; (**e**) plaintext histogram of Cameraman; (**f**) ciphertext histogram of Cameraman; (**g**) plaintext histogram of Rice; (**h**) ciphertext histogram of Rice.

The plaintext and ciphertext histograms of different images in the encryption and decryption process are shown in Figure 7.

As can be seen from Figure 7, the distribution ranges and intensities of pixels in the plaintext image histograms are uneven. After encryption using the algorithm in this paper, the pixel values in the ciphertext images are distributed within the range of 0 to 255, and the probability of occurrence of each pixel value is largely equivalent. This suggests that the statistical properties of the plaintext pixels have fundamentally changed. Therefore, the encryption algorithm proposed in this paper can effectively resist attacks based on statistical analysis.

#### 5.5.2. Analysis of Correlation between Adjacent Pixels

The correlation coefficients of adjacent pixels can be used to evaluate the effect of image encryption. In general, a relatively good digital image encryption scheme can result in relatively low correlation between adjacent pixels of a ciphertext image [7,54]. The closer to 0 the correlation coefficient of adjacent pixels is, the better the effect of encryption. In this paper, the correlation coefficients of adjacent pixels are calculated for 10,000 selected pairs of pixels in the images, and the calculation formula is

(18) $$\left\{ \begin{array}{l} \bar{x}=\frac{1}{N}\sum_{i=1}^{N}X_{i}\\ D\left(x\right)=\frac{1}{N}\sum_{i=1}^{N}{\left(x_{i}-\bar{x}\right)}^{2}\\ Conv\left(x,y\right)=\frac{1}{N}\sum_{i=1}^{N}\left(x_{i}-\bar{x}\right)\left(y_{i}-\bar{y}\right)\\ \gamma_{xy}=\frac{Conv\left(x,y\right)}{\sqrt{D\left(x\right)}\sqrt{D\left(y\right)}} \end{array}\right.$$

In Formula (18), the adjacent pixels in the image to be measured are expressed by *x* and *y*, respectively, while $\bar{x},\bar{y}$ are the average values of all x and y values. *N* pairs of pixels are selected, and $\gamma_{xy}$ is the correlation coefficient. To analyze the encryption algorithm proposed in this paper, 10,000 pairs of pixels are selected from the plaintext and ciphertext images of Lena, Lake, Cameraman, and Rice. For each plaintext and ciphertext image, correlation coefficients of adjacent pixels are calculated from the horizontal, vertical, and diagonal directions, with the results summarized in Table 5.

**Figure 8:**
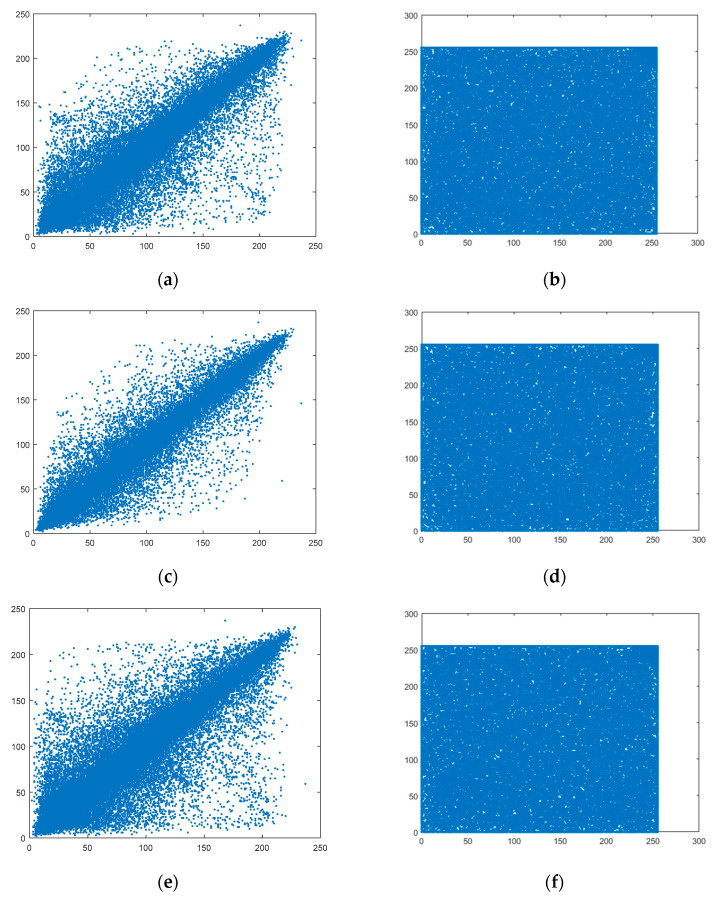
Correlation analysis of plaintext image and ciphertext image; (**a**) Lena-horizontal; (**b**) Encrypted image-horizontal; (**c**) Lena-vertical; (**d**) Encrypted image-vertical; (**e**) Lena-diagonal; (**f**) Encrypted image-diagonal.

As can be seen from Table 5, the correlation coefficients of adjacent pixels in plaintext images from all three directions are greater than 0.9, suggesting a high correlation degree between adjacent pixels. The correlation of adjacent pixels in the images encrypted by the algorithm proposed in this paper approaches 0. Taking Lena as an example, 10,000 pairs of pixels are selected from the original image and encrypted image among the horizontal, vertical, and diagonal directions. The gradation value relationships of adjacent pixels are shown in Figure 8.

As can be seen from Figure 8, the gray values of horizontal, vertical, and diagonal adjacent pixels from the plaintext image are distributed around *y = x*, while those from the ciphertext image are randomly distributed between 0 and 255.

#### 5.5.3. Information Entropy Analysis

In image encryption analysis, information entropy is the main parameter used for analyzing the randomness of information distribution in encrypted images [5]. The more random (uniform) the gray value distribution is, the greater the information entropy, and the better the effect of image encryption; conversely, the weaker the randomness, the lower the information entropy is. The information entropy *H* can be expressed as

(19) $$H_{m}=-\sum_{1}^{256}P(m_{i})\log_{2}P(m_{i})$$

In Formula (19), $P(m_{i})$ represents the probability of $m_{i}$ appearing in the image m. In this paper, the 8-bit Lena image is selected, with an ideal information entropy value of $H_{m}=8$. In 8-bit digital image analysis, the more random the encrypted image is, the closer the information entropy is to 8. The information entropies of plaintext and ciphertext images are listed in Table 6.

In Table 7, the information entropy of the ciphertext Lena image with a size of 256 × 256 obtained in this paper is compared with that from other studies [55,56]. As can be seen from the data, the algorithm proposed in this paper has achieved good results under the premise of data compression to save storage space. The information entropy of the plaintext image is relatively low, but the information entropy is very close to 8 after encryption.

### 5.6. Analysis of Resistance to Differential Attacks

The resistance of an encryption algorithm to differential attacks can be used to assess the sensitivity of a plaintext image. If a small change in the plaintext image does not result in a significant change in the ciphertext image, then the encryption algorithm is not resistant to differential attacks. If it results in a significant change, then the encryption algorithm can resist differential attacks. The resistance to differential attacks targeting an algorithm can be evaluated by calculating the pixel change rate (NPCR) and the unified average changing intensity (UACI) [57]. The formulae are as follows:

(20) $$NPCR=\frac{1}{m\times n}\sum_{i=1}^{m}\sum_{j=1}^{n}D(i,j)\times 100\%$$

(21) $$D(i,j)=\left\{ \begin{array}{l} 0,C_{1}\left(i,j\right){=C}_{2}\left(i,j\right),\;\\ 1,C_{1}\left(i,j\right)\neq C_{2}\left(i,j\right). \end{array}\right.$$

(22) $$UACI=\frac{1}{m\times n}\sum_{i=1}^{m}\sum_{j=1}^{n}\frac{\left|C_{1}\left(i,j\right)-C_{2}\left(i,j\right)\right|}{255}\times 100\%$$

In Formulas (20) and (21), *m* × *n* indicates the size of the image. $C_{1}(i,j)$ and $C_{2}(i,j)$ respectively indicate the pixel value of point (i,j) in the corresponding encrypted images of two plaintext images with only one different pixel. When the two plaintext images are only different by one pixel, the pixel value of point (i,j) is represented in the two ciphertext images as $C_{1}(i,j)$ and $C_{2}(i,j)$.

The resistance to a differential attack is tested in the simulation experiment with the grayscale images of Lena, Lake, Cameraman, and Rice, each of size 256 × 256. The average NPCR and UACI values of the algorithm proposed in this paper are listed in Table 8. The ideal values of NPCR and UACI are 99.6093% and 33.4635%, respectively [10].

It can be seen from the experimental results that the NPCR values and UACI values obtained by the encryption algorithm proposed in this paper are very close to the ideal values, indicating that the proposed algorithm has strong resistance to differential attacks.

## 6. Conclusions

In this paper, a new three-dimensional chaotic system is proposed for image encryption. The core of the encryption algorithm is the combination of chaotic system and compressed sensing, which is practical and can complete compression and encryption simultaneously. The Lyapunov exponent, bifurcation diagram and complexity of the new three-dimensional chaotic system are analyzed. The performance analysis shows that the chaotic system has two positive Lyapunov exponents and high complexity. It is verified that the new three-dimensional chaos proposed in this paper is practical and can be used in encryption. In the encryption scheme, a new chaotic system is used as the measurement matrix for compressed sensing, and Arnold is used to scramble the image further. The results of the encrypted scheme are analyzed and evaluated from two aspects. First, analyze the effects of reconstructed images. The proposed method has better reconfiguration ability in the compressible range of the algorithm compared with other methods. Second, analyze the security of the encryption algorithm. The proposed encryption algorithm can resist various attacks from key space, statistical analysis, information entropy and differential analysis. Finally, the proposed encryption scheme has good encryption effect and image compression capability.

## Figures and Tables

**Table 1:** *ApEn* of different chaotic sequences.

Chaotic System	Input Parameters	*ApEn*
Logistic	N = 2000, m = 2, r = 0.2SD	0.4918
Henon	N = 2000, m = 2, r = 0.2SD	0.4699
Lorenz	N = 2000, m = 2, r = 0.2SD	0.3197
Ours	N = 2000, m = 2, r = 0.2SD	0.6932

**Table 2:** PSNRs (dB) under different compression encryption methods.

Images	*v*	Ref. [43]	Ref. [44]	Ref. [45]	Ours
Lena(256×256)	0.75	29.56	30.82	30.21	33.25

**Table 3:** NIST test results.

Statistical Test	*p*-Value	Result
Frequency	0.843512	Passed
Block Frequency	0.697188	Passed
Cumulative Sums	0.593463	Passed
Runs	0.689301	Passed
Longest Run	0.314464	Passed
Rank	0.894036	Passed
FFT	0.421210	Passed
Non-Overlapping Templates	0.904121	Passed
Overlapping Templates	0.013027	Passed
Universal	0.301746	Passed
Approximate Entropy	0.693216	Passed
Random Excursions	0.011393	Passed
Random Excursions Variant	0.020299	Passed
Serial	0.498839	Passed
Linear Complexity	0.393688	Passed

**Table 4:** Key space comparison.

Algorithm	This Paper	Ref. [51]	Ref. [52]	Ref. [10]	Ref. [53]
Key space	2^624^	2^128^	2^140^	2^256^	2^398^

**Table 5:** Correlation coefficients of adjacent pixels in encrypted images.

Direction	Lena		Lake		Cameraman		Rice	
	Plaintext	Ciphertext	Plaintext	Ciphertext	Plaintext	Ciphertext	Plaintext	Ciphertext
Horizontal	0.9376	0.0 0 33	0.9526	0. 0028	0.9318	0.0021	0.9214	0.0056
Vertical	0.9660	0.0027	0.89531	0.0112	0.9559	0.0098	0.9374	0.0031
Diagonal	0.9753	0.0014	0.9206	0.0038	0.9076	0.0015	0.8934	0.0109

**Table 6:** Information entropy of plaintext and ciphertext images.

Image	Lena	Lake	Cameraman	Rice
Plaintext image	7.5686	7.4644	7.0097	7.0115
Ciphertext mage	7.9975	7.9973	7.9972	7.9976

**Table 7:** Comparison of information entropy of different schemes using the Lena image.

Algorithm	Information Entropy
This paper	7.9975
Ref. [55]	7.9979
Ref. [56]	7.9973

**Table 8:** Average NPCR and UACI values using the proposed algorithm.

Image	NPCR (ideal: 99.6093%)	UACI (ideal: 33.4635%)
Lena	99.6154%	33.3526%
Lake	99.5890%	33.3848%
Cameraman	99.6017%	33.3361%
Rice	99.6109%	33.3746%
